# Association of Skipping Breakfast with Metabolic Syndrome and Its Components: A Systematic Review and Meta-Analysis of Observational Studies

**DOI:** 10.3390/nu17193155

**Published:** 2025-10-03

**Authors:** Bowen Yang, Linxi Lian, Kaijun Xing, Yangyang Cen, Yi Zhao, Yannan Zhang

**Affiliations:** 1 School of Public Health, Ningxia Medical University, Yinchuan 750004, China; yangbowen9991126@163.com (B.Y.); 13598526169@163.com (L.L.); xkj0420@163.com (K.X.); cenyy_0126@163.com (Y.C.); zhaoyi751114@hotmail.com (Y.Z.); 2 Ningxia Key Laboratory of Environmental Factors and Chronic Disease Control, Yinchuan 750004, China

**Keywords:** skipping breakfast, metabolic syndrome, abdominal obesity, hypertension, hyperlipidemia, hyperglycemia, systematic review, meta-analysis

## Abstract

**Objective:** Metabolic syndrome (MetS) represents a growing and significant public health burden worldwide. The evidence regarding whether skipping breakfast affects the development of MetS and its components remains inconsistent and uncertain. This study aimed to synthesize the best available evidence regarding the association between skipping breakfast and the risk of MetS and its components. **Methods:** This systematic review and meta-analysis was conducted in accordance with the PRISMA 2020 guidelines. We systematically searched the PubMed, Embase, Cochrane Library, and Web of Science databases from inception until May 2025. Two reviewers independently screened titles/abstracts and full texts, extracted data, and assessed the risk of bias. This review included cross-sectional and cohort studies on the association between breakfast skipping and the risk of MetS and its components. **Results:** Nine studies were included after quality evaluation by NOS. Pooled results from the meta-analysis revealed that skipping breakfast was significantly associated with an increased risk of MetS (OR: 1.10, 95% CI: 1.04–1.17) and its components—namely abdominal obesity (OR = 1.17, 95% CI 1.01–1.34), hypertension (OR: 1.21, 95% CI: 1.10–1.32), hyperlipidemia (OR: 1.13, 95% CI: 1.04–1.23), and hyperglycemia (OR = 1.26, 95% CI: 1.16–1.37). **Conclusions:** The meta-analysis demonstrated that skipping breakfast was significantly associated with an increased risk of MetS and its key components—abdominal obesity, hypertension, hyperlipidemia, and hyperglycemia. These findings highlight regular breakfast consumption as a potential modifiable factor for preventing and managing MetS and related cardiometabolic diseases.

## 1. Background

Metabolic Syndrome (MetS) is a group of metabolic disorders with insulin resistance and central obesity as the common pathophysiological basis [1]. It is typically diagnosed when at least three of the following five components are present: abdominal obesity, elevated fasting glucose, hypertension, elevated triglycerides, and low levels of high-density lipoprotein cholesterol(HDL-C) [2]. The presence of MetS signifies a synergistic interaction among its components, which collectively markedly elevates the risk of atherosclerotic cardiovascular disease and accelerates the progression toward more severe metabolic disorders, including overt cardiovascular events and complications of diabetes [3,4]. The global prevalence of MetS is on the rise, with recent rates recorded at 31.1% in China (2015–2017) and 34.7% in the United States (2015–2016) [5,6]. This escalating prevalence underscores an urgent need for identifying novel and modifiable risk factors. Many studies have explored the impact of specific foods and dietary patterns on these conditions, but the influence of specific meal frequencies, such as breakfast, remains unclear.

Breakfast is widely regarded as the most important meal of the day contributing 20% to 35% of total daily energy intake [7]. A nutritionally balanced breakfast activates metabolic pathways and supports cognitive performance [8]. Long-term skipping breakfast or the imbalance of nutritional structure at breakfast pose significant health risks [9]. It can induce continuous deficiency of essential nutrients and interfere with energy metabolism homeostasis, leading to progressive deterioration of nutritional status [10]. At the same time, the central neurocognitive function is impaired, which is manifested as the decline of working memory capacity and executive control ability [11]. Moreover, epidemiological studies have confirmed that this behavior pattern independently increases the risk of hypertension and hyperglycemia by promoting insulin resistance, abnormal increase in sympathetic nerve tone and circadian rhythm disorder, and significantly increases the burden of cardiometabolic diseases [12].

A growing number of observational studies have investigated associations between breakfast skipping and the incidence or prevalence of MetS. However, evidence on the breakfast–MetS relationship is highly heterogeneous. Cross-sectional studies in South Korea reported positive associations [13], whereas another Korean cross-sectional analysis found none [14]. Moreover, a Japanese cohort study identified a sex-specific effect—risk was elevated in men but not in women [15]. The above conflicts of evidence highlight the methodological heterogeneity among studies, such as differences in population characteristics, confounding control, and exposure definition.

To date, no systematic review and meta-analysis has synthesized the available observational evidence on the relationship between skipping breakfast and MetS risk. Therefore, this systematic review and meta-analysis aimed to investigate the association between skipping breakfast (Exposure) and the prevalence/incidence of MetS and its components, including abdominal obesity, hypertension, hyperlipidemia, and hyperglycemia (Outcomes) among the general population (Population), compared with individuals who regularly consume breakfast (Comparison).

## 2. Materials and Methods

### 2.1. Protocol Registration

The following meta-analysis MOOSE epidemiological study (meta-analysis) guide [16] and PRISMA (system of choice for evaluation and meta-analysis report project) statement [17]. The study protocol was registered with PROSPERO (CRD420251027771), https://www.crd.york.ac.uk/PROSPERO/view/CRD420251027771 (accessed on 4 July 2025). We deviated from the protocol, restricting studies to English for accurate interpretation.

### 2.2. Search Strategy

We comprehensively searched the databases of PubMed, Embase, Cochrane, and the Web of Science. The search was limited to studies published in the English language. The search period was from the establishment of the databases to 7 April 2025. The electronic search was structured using a combination of keywords, medical subject headings (MeSHs), and Boolean operators (AND, OR) to refine the results. We used the following search criteria to find observational studies on potential dietary factors related to skipping breakfast and metabolic syndrome and its components: “skipping breakfast”, “breakfast frequency”, “breakfast”, “omitting breakfast”, “morning meal”, “fasting”, “metabolic syndrome”, “insulin resistance”, “obesity”, “blood pressure”, “blood glucose”, “hypertension”, “cholesterol”, “triglycerides”, “waist circumference”, “body mass index”, “low-density lipoprotein cholesterol”, “high-density lipoprotein cholesterol”, “total cholesterol”, “glycated hemoglobin”, “insulin resistance index”, “lipid profile”. The search was conducted using logical operators. Overall, these measures fall into 4 categories: anthropometric measures, lipid profile, blood pressure, and glycemic control measures. The limited number of relevant trials did not set limits. The whole string of the search is provided in the Supplementary Materials Section S1.

### 2.3. Inclusion Criteria

Studies were included if they met the following criteria: participants were from the general population without age restriction; the exposure of interest was the frequency of breakfast consumption; the outcome included a diagnosis of MetS or its individual components, based on a standard definition; the study design was observational, specifically cross-sectional, case–control, or cohort studies; and the study provided multivariate-adjusted effect estimates—such as odds ratios, risk ratios, or hazard ratios—along with their 95% confidence intervals for the association between breakfast consumption frequency and MetS or its components.

### 2.4. Exclusion Criteria

Studies were excluded if they were non-observational in design, focused on specific clinical populations (such as patients with diabetes), did not define breakfast frequency as the exposure, failed to provide multivariate-adjusted effect estimates with 95% confidence intervals, represented duplicate publications, were not published in English, or did not use MetS or its components as a defined outcome.

### 2.5. Data Extraction and Quality Assessment

Two investigators independently extracted data from all included studies. The extracted characteristics encompassed first author, publication year, study design, study period, study objective, country, sex and age of participants, sample size, definition of breakfast exposure, and reported outcome measures.

The methodological quality of the included observational studies was assessed using the Newcastle–Ottawa Scale (NOS), which is recommended by the Agency for Healthcare Research and Quality (AHRQ) for evaluating non-randomized studies [18]. The NOS judges study quality across three domains: selection of study groups, comparability of groups, and ascertainment of either the exposure or outcome [19]. A semi-quantitative “star system” is used to rate studies, with a maximum possible score of 9 stars. In accordance with established classification criteria [20], studies scoring ≥7 stars were deemed “high quality,” those with 4–6 stars “moderate quality,” and those with <4 stars “low quality.”

The quality assessment was performed independently by two reviewers. Any discrepancies in ratings were resolved through discussion until a consensus was reached. In cases where consensus could not be achieved, a third reviewer was consulted to make a final determination.

### 2.6. Eligibility for Synthesis

A systematic process was undertaken to decide the eligibility of included studies for each outcome-specific synthesis. Studies were categorized for synthesis based on the outcome type (MetS, abdominal obesity, hypertension, hyperlipidemia, hyperglycemia) and its diagnostic criteria. We extracted the operational definition of “skipping breakfast” and the detailed diagnostic criteria for each outcome from all studies. A comparative table of study characteristics was then created to match each study against the pre-specified synthesis groups. For the MetS synthesis, we included studies that used internationally recognized criteria. Studies applying other criteria were included in sensitivity analyses or reported separately. A single study could be included in multiple syntheses if it reported data on different outcomes. This categorization process was conducted independently by two reviewers, with disagreements resolved through discussion or by a third reviewer.

### 2.7. Statistical Methods

All studies included in this meta-analysis were pooled analyses using the estimated overall effect size statistic as the log of the observed OR (approximated to RR when necessary) because observed event rates were generally low and some raw data were incomplete. Cochran’s q test [21] was used to test whether there were significant statistical differences among the studies included in the meta-analysis, so as to reflect the heterogeneity of research results. When this index exceeds 50%, it is generally assumed that there is considerable heterogeneity between studies. Low heterogeneity was assumed to be *I*^2^ < 25% and moderate heterogeneity was assumed to be *I*^2^ = 25% to 50%. If there was no statistical heterogeneity between studies (*p* > 0.1, *I*^2^ < 50%), a fixed effects model should be used. Otherwise, random effects models should be used. To explore heterogeneity causes, we performed subgroup analyses by outcome criteria. Sensitivity analysis was used to explore possible sources of heterogeneity. Sensitivity analysis was performed by excluding each included study in turn, and then recalculating the combined effect size to observe whether the results had changed significantly. If the direction and significance of the overall results changed significantly after excluding individual studies, it may indicate that the study had a significant impact on the overall results, suggesting that there may be some sensitivity of the results. Forest plots were drawn to clearly visualize the synthesized association. The funnel plot technique was used to analyze the publication bias of observational studies. All statistical analyses were performed using Review Manager (RevMan) software version 5.4.1 developed by the Cochrane Collaboration. All tests were two-sided, and statistical significance was defined as *p* < 0.05.

## 3. Result

### 3.1. Research to Determine

**Figure 1:**
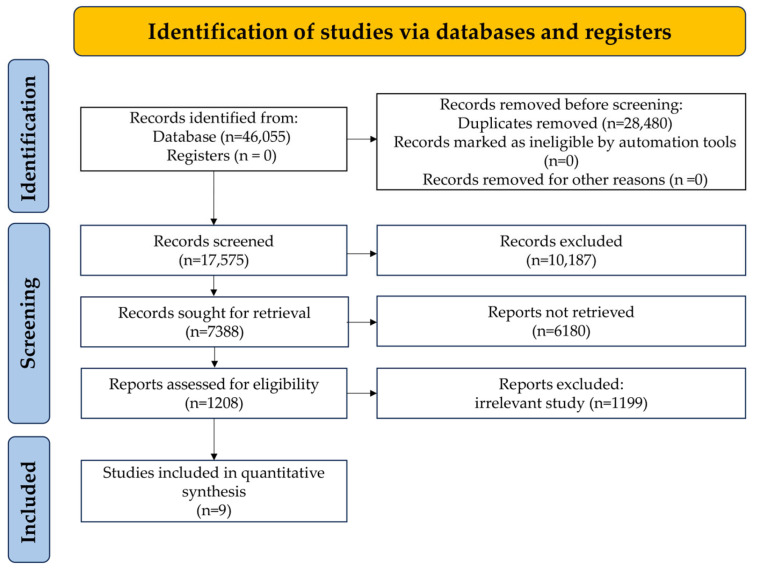
PRISMA Flowchart of Study Selection.

Inclusion process flowchart (Figure 1) illustrates the process of selecting the research. The initial database search yielded 45,432 publications. After screening by title and abstract, 10,187 records were excluded because they did not fit the theme or were duplicated. From the remaining 1208 publications, after careful reading and application of inclusion/exclusion criteria, 9 studies were finally included that met the criteria. These studies from Korea, Japan, the United States and other places included eight cross-sectional studies and two comparative studies. The characteristics of the included studies are summarized in Table 1. The quality of the research was evaluated using NOS. The included research scores were greater than 6 points, indicating that the research quality was medium or above (Table 1). The specific diagnostic criteria for MetS and its components used in each included study were provided in Supplementary Table S1.

### 3.2. Association Between Skipping Breakfast and Risk of MetS

A total of six studies, including five cross-sectional studies from Korea, Japan, the United States, and Iran [13,14,15,22,23] and one Japanese cohort study [24], explored the association between skipping breakfast and MetS risk. The results were inconsistent: two studies [23,24] identified skipping breakfast as a risk factor for MetS, while the other four [13,14,15,22] found no significant association.

**Figure 2:**
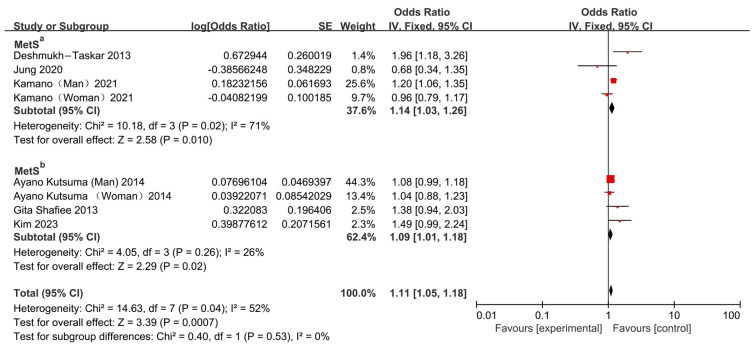
Forest plots were used to analyze the association between breakfast skipping and MetS [13,14,15,22,23,24]. MetS^a^: Metabolic syndrome defined using elevated fasting glucose values and/or a confirmed clinical diagnosis of type 2 diabetes as the glycemic criterion; MetS^b^: Metabolic syndrome defined using elevated fasting glucose values only as the glycemic criterion.

Subgroup analyses were performed based on the glycemic criterion used to define MetS (Figure 2). In the MetS^a^ subgroup (studies defining MetS with a glycemic criterion that encompassed either elevated fasting glucose or type 2 diabetes), skipping breakfast was significantly associated with an increased risk of MetS (OR = 1.14, 95% CI: 1.03–1.26). However, considerable heterogeneity was observed among these three studies (*I*^2^ = 71%, *p* = 0.02). In the MetS^b^ subgroup (studies defining MetS using elevated fasting glucose alone as the glycemic criterion), skipping breakfast was also associated with a significant increase in MetS risk (OR = 1.09, 95% CI: 1.01–1.18).

**Figure 3:**
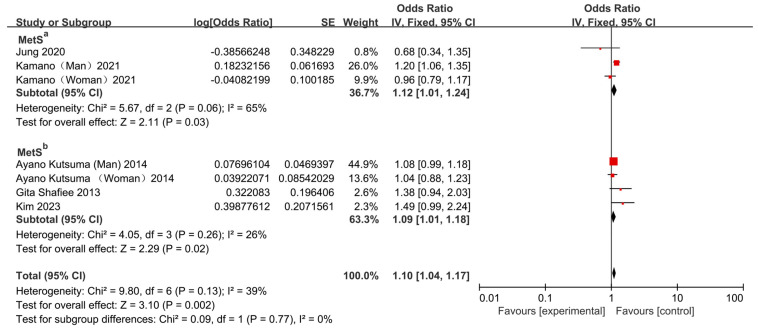
Forest plots were used to analyze the association between breakfast skipping and MetS after removal of heterogeneous studies [13,14,15,22,24]. MetS^a^: Metabolic syndrome defined using elevated fasting glucose values and/or a confirmed clinical diagnosis of type 2 diabetes as the glycemic criterion; MetS^b^: Metabolic syndrome defined using elevated fasting glucose values only as the glycemic criterion.

The overall pooled analysis demonstrated that skipping breakfast significantly increased the risk of MetS (OR = 1.11, 95% CI: 1.05–1.18; Figure 2), albeit with moderate heterogeneity (*I*^2^ = 52%, *p* = 0.04). Sensitivity analysis indicated that the study by Deshmukh-Taskar et al. (2013) [23] was the principal source of heterogeneity. After excluding this study, the association remained significant (OR = 1.10, 95% CI: 1.04–1.17; Figure 3).

### 3.3. Association Between Skipping Breakfast and Risk of MetS Components

#### 3.3.1. Association Between Skipping Breakfast and Risk of Abdominal Obesity

**Figure 4:**
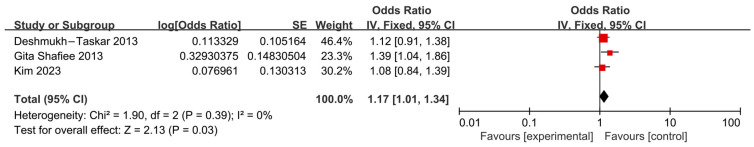
Forest plots were used to analyze the association between skipping breakfast and abdominal obesity [13,22,23].

Three cross-sectional studies conducted in Japan, the United States and Iran explored the association between missing breakfast intake and abdominal obesity [13,22,23]. Studies from Japan [13] and the United States [23] showed consistent null findings, with no statistically significant association observed between skipping breakfast and abdominal obesity risk. Whereas in Gita Shafiee’s [22] study, significant association observed between skipping breakfast and abdominal obesity risk. The pooled data from all three studies indicated that, overall, skipping breakfast is associated with an increased risk of abdominal obesity (OR = 1.17, 95% CI: 1.01–1.34; Figure 4).

#### 3.3.2. Association Between Skipping Breakfast and Risk of Hypertension

**Figure 5:**
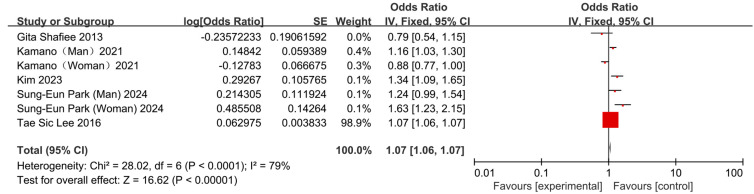
Forest plots of the association between skipping breakfast and hypertension [13,22,24,25,26].

**Figure 6:**
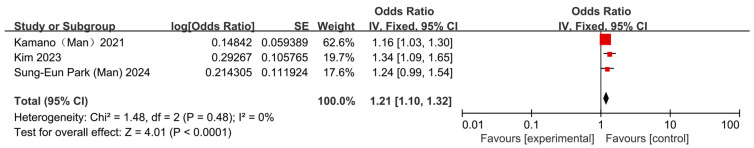
Forest plot of the association between skipping breakfast and hypertension after removal of heterogeneous studies [13,24,26].

Four cross-sectional studies and one cohort study [13,22,24,25,26] analyzed the association between skipping breakfast and hypertension risk. One study reported no significant association [22] while two others reported significant association [13,25]. The remaining two studies, however, reported sex-specific risks: Kamano et al. observed an increased risk in men but a protective effect in women [24], while Park et al. found an elevated risk in both sexes [26]. In the pooled analysis, skipping breakfast was significantly associated with an increased risk of hypertension (OR = 1.07, 95% CI: 1.06–1.07); however, notable heterogeneity was present (*I*^2^ = 79%, *p* < 0.05) (Figure 5). Sensitivity analysis indicated that Kamano (Women) (2021) [24], Sung-Eun Park (Woman) (2024) [26], Gita Shafiee (2013) [22] and Tae Sic Lee (2016) [25] were the principal sources of the heterogeneity. After accounting for these differences, the association remained significant (OR = 1.21, 95% CI: 1.10–1.32; Figure 6).

#### 3.3.3. Association Between Skipping Breakfast and Risk of Hyperglycemia

**Figure 7:**
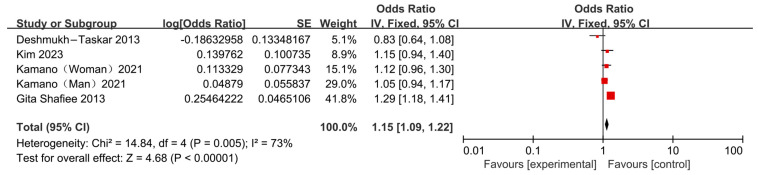
Forest plots of the association between skipping breakfast and hyperglycemia [13,22,23,24].

**Figure 8:**
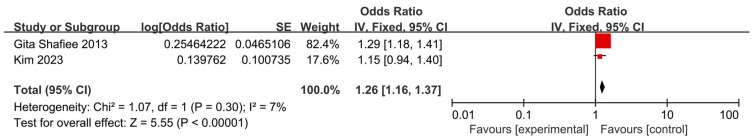
Forest plots of the association between skipping breakfast and hyperglycemia after removal of heterogeneous studies [13,22].

Three cross-sectional studies and one cohort study conducted in Japan, the United States and Iran explored [13,22,23,24] the association between skipping breakfast and risk of hyperglycemia. The results were heterogeneous: three studies [13,23,24] found no significant association between skipping breakfast and hyperglycemia. In contrast, the studies by Shafiee et al. [22] indicate that skipping breakfast was associated with an increased risk of hyperglycemia. The meta-analysis of these four studies showed that skipping breakfast significantly increased the risk of hyperglycemia (OR = 1.15, 95% CI: 1.09–1.22; Figure 7); however, substantial heterogeneity was observed (*I*^2^ = 73%, *p* = 0.005) (Figure 7). Sensitivity analysis indicated that Kamano (Women) (2021) [24], Kamano (Man) (2021) [24], and Deshmukh-Taskar (2013) [23] were the primary sources of heterogeneity. After accounting for these differences, the association remained significant (OR = 1.26, 95% CI: 1.16–1.37; Figure 8).

**Table 1:** Characteristics of the studies included in the systematic review.

First Author	Publication Year	Study Design	Country	Male (%)	Age (Years)	Study Objective	Study Population	Outcome (%)	Frequency of Breakfast	OR (95% CI)	Quality Score
Jung [14]	2020	cross-sectional study	Korea	49.1	20–64	Evaluate the influence of skipping breakfast on the MetS.	3864	MetS^a^: 8.5	0 times/week 1–4 times/week 5–7 times/week	0.68 (0.345–1.351)	9
Kamano [24]	2021	cohort study	Japan	50.06	35–69	Investigate sex-specific associations of skipping breakfast and short sleep duration with MetS and their interaction.	29,780	MetS^a^: 33.1 HTN: 87.5 (M); 87.9 (F) hyperglycemia: 77.7 (M); 69.5 (F) HLP: 68.6 (M); 55.5 (F)	Skipping breakfast	MetS^a^: 1.20 (1.06–1.35) (M) 0.96 (0.79–1.17) (F) HTN: 1.16(1.03–1.30) (M) 0.88 (0.77–1.00) (F) hyperglycemia: 1.05 (0.94–1.17) (M) 1.12 (0.96–1.30) (F) HLP: 1.18 (1.05–1.32) (M) 0.98 (0.82–1.18) (F)	9
Kutsuma [15]	2014	cross-sectional study	South Korea	62.7	20–75	Assess the association of breakfast skipping with MetS, proteinuria, obesity, and other cardiometabolic risk factors	54,155	MetS^b^: 12.1	Skipping breakfast	1.08 (0.99–1.19) (M) 1.04 (0.88–1.23) (F)	8
Kim [13]	2023	cross-sectional study	Japan	53.4	18–39	Assess the association between breakfast frequency and MetS.	12,302	MetS^b^: 2.6 AO: 9.1 HTN: 9.8 hyperglycemia: 10.2 HLP: 8.3	Non-skipping,4–6 days, and 0–3 days	1.49 (0.99–2.23) 1.08 (0.84–1.40) 1.34 (1.09–1.65) 1.15 (0.95–1.41) 1.09 (0.87–1.37)	8
Deshmukh-Taskar [23]	2013	cross-sectional study	USA	26.1	-	Examine the association between breakfast skipping and type of breakfast consumed with overweight/obesity, abdominal obesity, other cardiometabolic risk factors and MetS.	5316	MetS^a^: 20.7 AO: 36.8 HTN: 20.3 Hyperglycemia: 21.7	Skipping breakfast, ready-to-eat cereal	1.38 (0.94–2.03) 1.12 (0.95–1.38) 1.21 (0.98–1.48) 1.29 (0.95–1.14)	9
Tae Sic Lee [25]	2016	cross-sectional study	Korea	39.4	14–68	Investigate the relationship between the habit of eating breakfast and hypertension.	3880	HTN: 25.9	Skipping breakfast	1.065 (1.057–1.073)	7
Sung-Eun Park [26]	2024	cross-sectional study	Korea	30.3	19–64	Examine the association of breakfast habits with hypertension and obesity risk.	2779	HTN: 13.7	Regular breakfast, skip breakfast every day	1.239 (0.995–1.543) (M) 1.625 (1.228–2.148) (F)	7
Gita Shafiee [22]	2013	cross-sectional study	Iran	-	10–18	Evaluate the association of breakfast intake with cardiometabolic risk factors.	5625	MetS^b^: 5.25 HLP: 9.76 AO: 19.61 HTN: 4.89 Hyperglycemia: 14.02	None, 1–2 days, 3–6 days, every day	1.96 (1.18–3.27) 1.41 (1.03–1.93) 1.39 (1.04–1.86) 0.79 (0.54–1.14) 0.83 (0.64–1.08)	8
Fabiana A Silva [27]	2018	cross-sectional study	Brazil	-	7–14	Investigated the demographic, anthropometric, clinical, biochemical and behavioral factors associated with populations who missed breakfast.	684	HLP: 11.4	Skipping breakfast	0.79 (0.29–2.15)	8

MetS: Metabolic Syndrome; M: Male; F: Female; AO: Abdominal Obesity; HTN: Hypertension; HLP: Hyperlipidemia; MetS^a^: Metabolic syndrome defined using elevated fasting glucose values and/or a confirmed clinical diagnosis of type 2 diabetes as the glycemic criterion; MetS^b^: Metabolic syndrome defined using elevated fasting glucose values only as the glycemic criterion.

#### 3.3.4. Association Between Skipping Breakfast and Risk of Hyperlipemia

**Figure 9:**
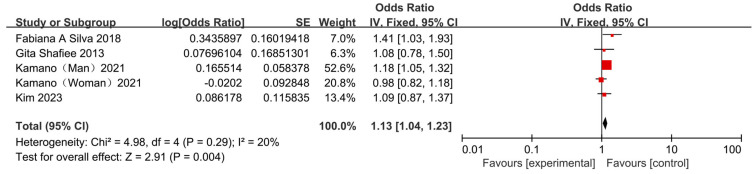
Forest plots of the association between skipping breakfast and hyperlipidemia [13,22,24,27].

Three cross-sectional observational studies and one cohort study based on Japanese, Iranian, and Brazilian populations [13,22,24,27] examined the association between skipping breakfast and hyperlipemia. The results across studies were heterogeneous. Two studies found no significant association between skipping breakfast and hyperlipidemia [13,22]. However, Fabiana A. Silva’s study found that skipping breakfast significantly increased the risk [27]. In contrast, Kamano et al. observed a more complex, sex-dependent effect—an increased risk in men but a protective effect in women [24]. Meta-analysis of above four studies showed that the skipping breakfast significantly increases the risk of hyperlipemia (OR = 1.13, 95% CI: 1.04–1.23, Figure 9).

## 4. Discussion

This systematic review and meta-analysis, which synthesized evidence from nine observational studies involving 118,385 participants, provides compelling evidence that skipping breakfast is significantly associated with an increased risk of MetS and its key components. Our pooled results demonstrate that individuals who regularly skip breakfast have a higher prevalence or incidence of MetS, along with elevated risks of abdominal obesity, hyperglycemia, dyslipidemia, and hypertension. These findings underscore the importance of breakfast consumption as a modifiable dietary behavior that may play a critical role in the prevention of metabolic disorders. The consistency of these associations across diverse populations and study designs strengthens the plausibility of a causal relationship, although residual confounding cannot be fully excluded.

### 4.1. Skipping Breakfast Increase MetS Risk

This meta-analysis shows that skipping breakfast significantly increases the risk of MetS. The pooled analysis showed that people who skipped breakfast had 1.10 times the risk of MetS compared with those who ate breakfast. This finding is consistent with broader epidemiological evidence highlighting the importance of breakfast habits for metabolic health. For instance, larger breakfast size (energy intake) has also been associated with a lower risk of MetS, further supporting the concept that breakfast consumption is a significant dietary factor in metabolic health [28]. Skipping breakfast can disrupt the circadian rhythm and metabolic homeostasis. The human body follows a natural rhythm, and skipping breakfast breaks this rhythm, leading to metabolic regulation disorders and affecting the body’s ability to process nutrients [29]. Patients with MetS should choose a regular and healthy diet, stick to having breakfast and avoid prolonged fasting [30].

### 4.2. Skipping Breakfast and Abdominal Obesity

Our meta-analysis provides robust evidence that skipping breakfast is significantly associated with an elevated risk of abdominal obesity, a central component of MetS. This finding is biologically plausible, as breakfast omission may lead to prolonged post-absorptive states, compensatory overeating later in the day, and impaired insulin sensitivity, all of which can promote visceral fat accumulation. This aligns with a previous meta-analysis that reported skipping breakfast is associated with an increased risk of overweight/obesity in children and adolescents, suggesting this relationship may persist across the lifespan [31]. Although individual studies in our analysis showed inconsistencies—possibly due to variations in population characteristics or definitions—the pooled results clarify an overall positive association. This reinforces the importance of regular breakfast consumption as a potentially modifiable dietary behavior for preventing abdominal obesity and MetS.

### 4.3. Skipping Breakfast Increase Hypertension Risk

This meta-analysis shows that skipping breakfast significantly increases the risk of developing hypertension. Our findings are consistent with a 2022 meta-analysis of six observational studies (involving 14,189 participants, including 3577 breakfast skippers), which also reported a significant association between skipping breakfast and hypertension [32]. The pooled results showed that skipping breakfast was significantly associated with hypertension in these populations, which is consistent with the results of this study. Insulin resistance caused by skipping breakfast can stimulate sympathetic nervous system activity, promote renal sodium reabsorption, and lead to increased blood pressure [33]. Obesity and inflammation caused by skipping breakfast are also the main causes of high blood pressure [34].

### 4.4. Skipping Breakfast Increase Hyperglycemia Risk

The present meta-analysis provides compelling evidence that skipping breakfast is significantly associated with an increased risk of hyperglycemia, a key component of MetS. This finding aligns with the overall conclusion of our study, which identified a detrimental link between breakfast omission and the clustering of cardiometabolic risk factors that define MetS.

The pathophysiological mechanisms underlying this association are likely multifactorial. Skipping breakfast can prolong the fasting period, and long-term fasting can lead to a drop in blood sugar [35]. However, when the body is in a state of hypoglycemia, it secretes more insulin during meals to cope with the intake of food, thereby quickly bringing blood sugar back to the normal range. This recurrence causes insulin resistance [36]. Furthermore, skipping breakfast often leads to overcompensation of energy intake later in the day, particularly through large, high-calorie meals. These pronounced postprandial glucose spikes can place a significant burden on pancreatic β-cell function and contribute to chronic elevations in blood glucose levels.

### 4.5. Breakfast Increase Hyperlipidemia Risk

Hyperlipidemia refers to a state in which the levels of lipids (mainly cholesterol and triglycerides) in the body are abnormally elevated [37]. It is also one of the important factors leading to cardiovascular diseases (such as coronary heart disease, stroke, myocardial infarction) [38]. This meta-analysis shows that skipping breakfast significantly increases the risk of hyperlipidemia. This is consistent with the findings of a meta-analysis in 2021, which found that skipping breakfast was significantly associated with hyperlipidemia in three studies (*n* = 8511) [39]. Skipping breakfast is associated with total cholesterol, low-density lipoprotein cholesterol and triglyceride levels, as well as lower HDL-C levels [40]. Insulin resistance caused by skipping breakfast, long fasting time leading to overeating at lunch, and prolonged postprandial hyperlipidemia [41].

### 4.6. Skipping Breakfast vs. Intermittent Fasting

Our meta-analysis found a significant adverse association between skipping breakfast and the risk of MetS. This finding, however, appears to contradict the growing body of literature documenting the metabolic benefits of various forms of intermittent fasting. It is therefore crucial to delineate the fundamental distinctions between these two dietary patterns to reconcile this apparent paradox.

The critical difference lies in the context and patterning of the fasting period. Skipping breakfast, as examined in our meta-analysis, typically represents an unstructured, uncontrolled eating pattern. It is often associated with other unhealthy lifestyle behaviors (e.g., overall poor diet quality, circadian rhythm disruption) and may lead to overcompensation of energy intake later in the day, promoting metabolic dysregulation [42]. In contrast, intermittent fasting is a structured dietary regimen that involves well-defined cycles of fasting and eating (e.g., 16:8 time-restricted feeding) [43]. It is practiced consciously, often within the context of an overall healthy diet and lifestyle. The metabolic benefits of intermittent fasting are hypothesized to stem from sustained, controlled periods of low insulin levels and the induction of cellular autophagy [44], which are not achieved with the irregular and compensatory eating patterns commonly seen in breakfast skippers.

### 4.7. Strengths and Limitations

This study has several noteworthy strengths: To reduce confounding bias and obtain more accurate results, we included a large number of participants and events from prospective cohort studies, most of which were conducted over an extended period of time. To ensure reliability, we also performed sensitivity analyses. This comprehensive meta-analysis establishes the association between skipping breakfast and risk of MetS and its components.

However, our findings should be interpreted in the context of several limitations. Firstly, due to the observational nature of the included studies, there may be some residual and unmeasured confounding (e.g., socioeconomic status, overall diet quality). Secondly, different methods of assessing both breakfast consumption and outcomes across the included studies may introduce measurement bias and affect the consistency of the results.

## 5. Conclusions

This meta-analysis showed that skipping breakfast significantly increased the risk of MetS, and its components, including abdominal obesity, hyperlipidemia, hypertension, and hyperglycemia. Given the highly modifiable nature of this causal relationship, public health strategies that include regular consumption of a well-balanced breakfast may be one of the most cost-effective lifestyle interventions for the prevention and management of cardiometabolic diseases, especially in high-risk populations.

## Data Availability

Not applicable.

## Supplementary Materials

The following supporting information can be downloaded at: https://www.mdpi.com/article/10.3390/nu17193155/s1, Section S1: The whole string for search. Table S1: Diagnostic criteria of metabolic syndrome and its components across the included studies.
