# Supplementary material for: Association of Skipping Breakfast with Metabolic Syndrome and Its Components: A Systematic Review and Meta-Analysis of Observational Studies
Source: Nutrients. 2025 Oct 3;17(19):3155. doi: 10.3390/nu17193155 (PMC12525555; doi:10.3390/nu17193155)
Supplement: Supplementary file 1 [file nutrients-17-03155-s001.zip › nutrients-3869018-supplementary.pdf]

Supplemental Materials

Section S1: The whole string for search

("Breakfast"[Mesh] OR "Skipping Breakfast"[Mesh] OR "breakfast skip*"[tiab] OR "skip* breakfast"[tiab] OR "omit* breakfast"[tiab] OR "breakfast omission"[tiab] OR "missed breakfast"[tiab] OR "morning meal" OR "fasting" OR "breakfast frequency" OR (breakfast* AND (skip* OR frequent* OR omit* OR omis* OR consum*)))
AND ("Metabolic Syndrome"[Mesh] OR "Insulin Resistance"[Mesh] OR "Obesity"[Mesh] OR "Blood Pressure"[Mesh] OR "Blood Glucose"[Mesh] OR "Cholesterol"[Mesh] OR "Triglycerides"[Mesh] OR "metabolic syndrome"[tiab] OR "insulin resistance"[tiab] OR "obesity"[tiab] OR "hypertension"[tiab] OR "blood glucose"[tiab] OR "cholesterol"[tiab] OR "triglyceride"[tiab] OR "weight" OR "body mass index (BMI)" OR "waist circumference" OR "waist-to-hip ratio (waist:hip ratio)" OR "lipid profiles" OR "high-density lipoprotein cholesterol (HDL-C)" OR "low-density lipoprotein cholesterol (LDL-C)" OR "total cholesterol (TC)" OR "total triglyceride (TG)" OR "diastolic blood pressure (DBP)" OR "systolic blood pressure (SBP)" OR "glucose" OR "blood glucose" OR "blood sugar" OR "fasting blood sugar (FBS)" OR "fasting plasma glucose (FPG)" OR "fasting blood glucose (FBG)" OR "glycated hemoglobin (HbA1c)" OR "insulin resistance index (HOMA-IR)")
AND ("randomized controlled trial"[pt] OR "clinical trial"[pt] OR "prospective study"[tw] OR "cohort study"[tw] OR "cross-sectional study"[tw] OR "cross sectional"[tw] OR "observational study"[tw] OR "case control study"[tw])

Table S1. Diagnostic criteria of metabolic syndrome and its components across the included studies

First author	Publication year	Study design	Outcome	Definition
Jung[1]	2020	Cross-sectional study	Metabolic syndrome	A diagnosis was posed when three or more criteria listed below were met:<br>1. Abdominal obesity: waist circumference ⩾90 cm (male) and ⩾80 cm (female)<br>2. Triglycerides ⩾150 mg/dL High density lipoprotein cholesterol (HDL): <40 mg/dL for men, <50 mg/dL for women<br>3. Fasting blood glucose ⩾100 mg/dL, insulin injection to treat diabetes, or taking oral hypoglycemic drugs<br>4. Blood pressure: systolic blood pressure/diastolic blood pressure ⩾ 130/85 mmHg or taking anti-hypertensive drugs
Kamano[2]	2021	Cohort study	Metabolic syndrome	MetS was diagnosed when participants had at least three of the following five conditions:<br>1. Obesity: BMI ≥25 kg/m^2 instead of high waist circumference;<br>2. High blood pressure: systolic blood pressure ≥130 mmHg and/or

				diastolic blood pressure ≥85 mmHg or receiving treatment for hypertension;<br>3. Elevated triglycerides: serum triglyceride level ≥150 mg/dL;<br>4. Low HDL cholesterol: serum HDL cholesterol level <40 mg/dL in men or <50 mg/dL in women; and<br>5. Elevated blood glucose: fasting plasma glucose level ≥100 mg/dL or receiving treatment for diabetes.
Kutsuma[3]	2014	Cross-sectional study	Metabolic syndrome	Because of the lack of fasting plasma glucose measurement, the diagnosis of MetS was based on the modified Adult Treatment Panel III criteria with the following cutoff limits:<br>1. systolic blood pressure ≥ 130 mmHg or diastolic blood pressure ≥ 85 mmHg (elevated blood pressure);<br>2. triglycerides ≥ 150 mg/dL; high-density lipoprotein cholesterol (HDLC) < 40 mg/dL for men and <50 mg/dL for women (low HDL-C);<br>3. HbA1c ≥ 5.6% (high-normal HbA1c); and<br>4. waist circumference ≥ 90 cm for men and ≥80 cm for women in consideration of ethnic difference.<br>Subjects meeting three or more of these criteria were defined as having MetS. Because fasting plasma glucose data were unavailable in this study, HbA1c ≥ 5.6% was used as a surrogate marker for elevated fasting plasma glucose of ≥100 mg/dL.
Kim[4]	2023	Cross-sectional study	Metabolic syndrome	The definition of metabolic syndrome was taken from the modified National Cholesterol Education Program (NCEP) Adult Treatment Panel III guidelines19. Subjects who met three or more of the following criteria were diagnosed with metabolic syndrome:<br>1. central obesity (waist circumference≥90 cm for men or≥85 cm for women);<br>2. hypertriglyceridemia with fasting plasma triglyceride levels≥ 150 mg/dL;<br>3. decreased levels of high density lipoprotein cholesterol (HDL-C) with HDL-C levels < 40 mg/dL for men and < 50 mg/dL for women;<br>4. hypertension with systolic or diastolic BP ≥ 130/85 mmHg; and<br>5. hyperglycemia with fasting plasma glucose≥100 mg/dL.
Deshmukh-Taskar[5]	2013	Cross-sectional study	Metabolic syndrome	The MetS was defined using the American Heart Association/National Heart, Lung, and Blood Institute Adult Trea, i.e. having three or more of the following risk factors:<br>1. abdominal obesity, WC≥ 102 cm (males) or WC≥ 88 cm (females);<br>2. elevated blood pressure, systolic blood pressure≥ 130 mmHg or diastolic blood pressure ≥ 85 mmHg or antihypertensive medication use;<br>3. reduced serum HDL-cholesterol, <40 mg/dL (males) or <50 mg/dL (females) or medication use for reduced HDL-cholesterol;<br>4. elevated serum TAG≥ 150 mg/dL or medication use for elevated TAG;<br>5. elevated fasting plasma or serum glucose≥100 mg/dL or medication

				use for elevated glucose.
Tae Sic Lee[6]	2016	Cross-sectional study	Hypertension	A systolic blood pressure (SBP) ≥140 mmHg or a diastolic blood pressure (DBP) ≥90 mmHg was classified as hypertension.
Sung-Eun Park[7]	2024	Cross-sectional study	Hypertension	Blood pressure categories were defined according to the guidelines of the American Heart Association and the Korean Society of Hypertension. A systolic blood pressure (SBP) ≥140 mmHg or a diastolic blood pressure (DBP) ≥90 mmHg was classified as hypertension. Participants with an SBP of 160 mmHg or higher, a DBP of 100 mmHg or higher, or those currently on antihypertensive treatment were excluded from the analysis.
Gita Shafiee[8]	2013	Cross-sectional study	Metabolic syndrome	Subjects were classified as having MetS if they had at least three of the following criteria according to Adult Treatment Panel III (ATP III) criteria modified for the pediatric age group.<br>1. abdominal obesity, WC at or above the 90th percentile value for age and sex;<br>2. elevated BP, either systolic or diastolic BP at or above the 90th percentile for age, sex and height;<br>3. low HDL-C, HDL-C ⩽ 40(< 50) mg/dL (except in boys of 15-19 years old in which the cut off was < 45 mg/dL);<br>4. high TG: TG ⩾ 100 mg/dL) was taken as the 90th percentile value for age;<br>5. high FBG---FBG levels of ⩾100 mg/dL.<br>High cholesterol and low-density lipoprotein cholesterol were defined according to the recent recommendations by the American Heart Association; i.e. total cholesterol ⩾ 200 mg/dL, LDL-C>110 mg/dL. The definition of generalized obesity was considered as BMI > 95th percentile. Abdominal obesity was defined as waist to height ratio (WHtR) more than 0.5.
Fabiana A Silva[9]	2018	Cross-sectional study	Hyperlipidemia	Changes in serum lipids were analysed using reference values for children and adolescents, according to the 1st Guideline for Preventing Atherosclerosis in Childhood and Adolescence and those with serum lipids above the threshold values were considered at risk. Fasting glucose was classified according to criteria established by the International Diabetes Federation.

References

[1] J. Jung, A.-S. Kim, H.-J. Ko, H.-I. Choi, and H.-E. Hong, "Association between Breakfast Skipping and the Metabolic Syndrome: The Korea National Health and Nutrition Examination Survey, 2017," *Medicina*, vol. 56, no. 8, p. 396, Aug. 2020, doi: 10.3390/medicina56080396.

[2] S. Katsuura-Kamano *et al.*, "Association of skipping breakfast and short sleep duration with the prevalence of metabolic syndrome in the general Japanese population: Baseline data from the Japan Multi-Institutional Collaborative cohort study," *Prev Med*

Rep, vol. 24, p. 101613, Oct. 2021, doi: 10.1016/j.pmedr.2021.101613.

[3] A. Kutsuma, K. Nakajima, and K. Suwa, "Potential Association between Breakfast Skipping and Concomitant Late-Night-Dinner Eating with Metabolic Syndrome and Proteinuria in the Japanese Population," *Scientifica*, vol. 2014, pp. 1–9, 2014, doi: 10.1155/2014/253581.

[4] H. M. Kim, H. J. Kang, D. H. Lee, S.-M. Jeong, and H.-K. Joh, "Association between breakfast frequency and metabolic syndrome among young adults in South Korea," *Sci Rep*, vol. 13, no. 1, p. 16826, Oct. 2023, doi: 10.1038/s41598-023-43957-3.

[5] P. Deshmukh-Taskar, T. A. Nicklas, J. D. Radcliffe, C. E. O'Neil, and Y. Liu, "The relationship of breakfast skipping and type of breakfast consumed with overweight/obesity, abdominal obesity, other cardiometabolic risk factors and the metabolic syndrome in young adults. The National Health and Nutrition Examination Survey (NHANES): 1999–2006," *Public Health Nutr*, vol. 16, no. 11, pp. 2073–2082, Nov. 2013, doi: 10.1017/S1368980012004296.

[6] T. S. Lee, J. S. Kim, Y. J. Hwang, and Y. C. Park, "Habit of Eating Breakfast Is Associated with a Lower Risk of Hypertension," *J Lifestyle Med*, vol. 6, no. 2, pp. 64–67, Sep. 2016, doi: 10.15280/jlm.2016.6.2.64.

[7] S.-E. Park, S.-Y. Roh, and S.-Y. Kim, "Relationship of Breakfast Habits with Hypertension and Obesity: A Cross-Sectional Study among Korean Adults and Older Adults," *ijph*, Jan. 2025, doi: 10.18502/ijph.v54i1.17589.

[8] G. Shafiee *et al.*, "Association of breakfast intake with cardiometabolic risk factors," *Jornal de Pediatria*, vol. 89, no. 6, pp. 575–582, Nov. 2013, doi: 10.1016/j.jped.2013.03.020.

[9] F. A. Silva *et al.*, "Cross-sectional study showed that breakfast consumption was associated with demographic, clinical and biochemical factors in children and adolescents," *Acta Paediatrica*, vol. 107, no. 9, pp. 1562–1569, Sep. 2018, doi: 10.1111/apa.14363.
